# Physical Characterization and Biodistribution of Cisplatin Loaded in Surfactant Modified-Hybrid Nanoparticles Using Polyethylene Oxide-b-Polymethacrylic Acid

**DOI:** 10.34172/apb.2021.086

**Published:** 2020-10-20

**Authors:** Andang Miatmoko

**Affiliations:** Department of Pharmaceutical Sciences, Faculty of Pharmacy, Universitas Airlangga, Nanizar Zaman Joenoes Building, Campus C Mulyorejo, Surabaya, 60115, Indonesia.

**Keywords:** Cisplatin, Cancer, Egg phosphatifylcholine, Hybrid nanoparticles, Polyethylene oxide-b-polymethacrylic acid, Surfactants

## Abstract

***Purpose:*** Conjugating cisplatin into hybrid nanoparticles is intended to enhance tumor accumulation in cancer therapy due to drug interaction with polymer and prevent premature drug release because of the presence of a lipid layer.

***Methods:*** Hybrid nanoparticles composed of polyethylene oxide-b-polymethacrylic acid, egg phosphatidylcholine, and surfactant, i.e. sodium cholate/sodium deoxycholate/Tween 80, were prepared by the injection method. Cisplatin was subsequently loaded by incubating the polymer-drug mixtures at the molar ratio of carboxylate ions of 2:1.

***Results:*** The results showed that the addition of surfactants produced particle sizes between 33 and 52 nm. The addition of cisplatin increased the ζ-potential to slightly positive charges with encapsulation efficiencies of 5%-18%. An *in vivo* biodistribution study of mice identified a cisplatin plasma concentration of sodium cholate-modified hybrid nanoparticles 10 times higher than cisplatin solution, thus producing high tumor accumulation.

***Conclusion:*** Conjugating cisplatin into sodium cholate-modified hybrid nanoparticles improves its accumulation in tumors.

## Introduction

Cisplatin, classified as a platinum complex alkylating-drug, has been widely employed in chemotherapy, either as a single-drug therapy or in combination with other chemotherapeutic agents.^1-4^ However, numerous reports exist regarding the severe side effects correlated to dosage and dose intensity of cisplatin therapy including nephrotoxicity,^5,6^ distal neuropathy,^7,8^ nausea, vomiting, and anorexia,^9-11^ ototoxicity,^12,13^ and liver toxicity.^14,15^

It has been reported that cisplatin has a biphasic pharmacokinetic profile with a first half-life of between 25-49 minutes and a second which lasts for 58-73 hours after intravenous injection.^11^ Moreover, cisplatin also has a high tissue-blood partition ratio in the kidney.^16^ Enhancing the circulation lifetime of drugs in the body by drug conjugation into nanoparticles may improve the efficacy of cisplatin in addition to reducing its toxicity. Particulate carriers such as polymeric micelles, liposomes, nanoparticles, and many others have been investigated in order to achieve higher drug accumulation in tumors and reduce distribution to healthy tissues and organs during cisplatin therapy.^17-22^

Hybrid nanoparticles, which combine the properties of lipid vesicles and polymeric micelle, are potentially excellent drug carriers. Generally, hybrid nanoparticles contain diblock polymer and lipid components. The diblock polymer itself possesses two different functional block segments that can generate polymeric micelles. In addition, the lipid layer surrounding the core serves as a highly biocompatible-biomimetic shell and water diffusion constraint.^23,24^ This lipid layer may also support and protect the inner layer from premature disintegration caused by excessive dilutions upon systemic administration.^25^ A previous study reported that hybrid nanoparticles enhance the stability of cisplatin-polymer conjugation and result in superior *in vitro* stability to polymeric micelle.^26^ However, it indicated no improved levels of cisplatin in tumor tissue. Therefore, hybrid nanoparticle-loading cisplatin was modified by the addition of a surfactant whose presence affects lipid deformability.^27,28^

In this study, a biodegradable block ionomer, polyethylene oxide-b-polymethacrylic acid (PEO-b-PMAA), was used to prepare the hybrid nanoparticles. PEO is a relatively non-toxic water soluble non-ionic homopolymer^29^ which demonstrates the ability to maintain the recognition of nanoparticles by the immune system providing longer drug circulation in plasma.^30,31^ PMAA is a pH-dependently ionized weak anionic polymer which will conjugate with the platinum ions, together forming the inner core and controlling the drug release specific to the acidic tumor environment via reversible ion exchanges.^32^ Given the presence of abundant counterions, the drug-polymer interaction will cease leading to the disintegration of the micelle, thus releasing the encapsulated drug.^33^ The addition of lipid such as egg phosphatidylcholine and surfactant, as the hybrid nanoparticle component, is intended to enhance the stability of nanoparticles as well as high tumor drug accumulation. Surfactant has been known to destabilize the lipid bilayer, thereby altering the membrane’s permeability and flexibility.^34-37^ The use of surfactant may affect the drug released from the carrier resulting in changes to the drug levels in plasma and tumor tissue. Conjugating cisplatin into hybrid nanoparticles was intended to enhance the stabilization of polymer-drug interaction during blood circulation, thus promoting tumor drug accumulation.

## Materials and Methods

### Materials

Cisplatin was purchased from Wako Pure Chemical Industries Co., Ltd. (Tokyo, Japan). Egg phosphatidylcholine (EPC, Coatsome^®^ NC-50) and Tween 80 were products of NOF Inc. (Tokyo, Japan). Sodium Deoxycholate (SD) was acquired from Wako Pure Chemical Industries Co., Ltd. (Osaka, Japan). Sodium cholate (SC) was supplied by Sigma Aldrich (Tokyo, Japan). The diblock polymer used in this experiment was polyethylene oxide-b-polymethacrylic acid (PEO-b-PMAA; M_w_ of PEO = 7500; M_n_ of PMAA= 11 000) was obtained from Polymer Source, Inc. (Canada). Saline was purchased from Otsuka Co. Ltd. (Japan). In order to undertake high-performance liquid chromatography (HPLC) analysis, all solvents were of HPLC analytical grade. For graphite furnace atomic absorption spectrophotometry (GF-AAS) measurements, nitric acid (1.38; analytical grade, Wako Pure Chemical Industries Co., Ltd., Osaka, Japan) was employed as a digestive acid solution, while hydrochloric acid (AAS analytical grade) was used for the sample solvent (Kanto Chemical Co., Inc., Tokyo, Japan). The solvents; ethanol, methanol, acetone, and chloroform; were purchased from Wako Pure Chemical Industries, Ltd. (Osaka, Japan). All other reagents and solvents employed in this study were of the highest quality available. Milli-Q water was used in all experiments.

### Preparation of cisplatin hybrid nanoparticles

Firstly, the matrix of hybrid nanoparticles was prepared by injection method.^38^ The diblock polymer, PEO-b-PMAA, was dissolved in methanol, while EPC, sodium deoxycholate, sodium cholate and Tween 80 were dissolved in methanol. As seen from the contents of Table 1, appropriate amounts of EPC and each surfactant were mixed by vortexing, with the polymer solution subsequently being added and agitated until it was homogenous. The solution was quickly injected into the water and left at room temperature. In order to remove the organic solvents, approximately 20 mL of the mixture was dialyzed with 200 mL of water using a regenerated cellulose dialysis membrane (Spectra Por^®^7) with a molecular weight cut-off (MWCO) of 2000. The water was changed a total of eight times on two consecutive days.

**Table 1:** Molar compositions of hybrid nanoparticles prepared with different surfactants

**Formula**	**Molar Ratio**		
	**EPC**	**Surfactant (SD, SC, TW)**	**PEO-b-PMAA**
HNP	50	-	2.8
HNP-SD	50	5.0	2.8
HNP-SC	50	5.0	2.8
HNP-TW	50	5.0	2.8

Cisplatin was incorporated into the hybrid nanoparticles by direct mixing of cisplatin solution in an alkaline condition with a molar ratio of carboxylate ions [COO-]:[cisplatin] of 2:1.^33^ The carboxylate molar concentration was determined by acid-base titration method. The pH of the mixture was adjusted to pH 9 by means of an ammonia solution and it was then incubated for two days in a shaking water bath at 37°C. The hybrid nanoparticle-loaded cisplatin was obtained by filtering the mixtures with a centrifugal filter unit (Merck Millipore Ltd., Carrigtwohill, Ireland) with an MWCO of 30,000 at 2,500 G for 20 minutes. The filtrate was further used to determine encapsulation efficiency.

### Measurement of particle size, ζ-potential, entrapment efficiency, and loading capacity of cisplatin hybrid nanoparticles

The average particle size and ζ-potential of cisplatin-loaded hybrid nanoparticles was measured by cumulant method and electrophoretic mobility with a light scattering photometer (ELS-Z2, Otsuka Electronics Co., Ltd., Osaka, Japan) at 25°C. The undiluted samples were subsequently measured directly.

The entrapment efficiency was determined by calculating the cisplatin content of the hybrid nanoparticles. As previously reported, the sample extraction was performed using the Bligh and Dyer method.^26,39^ Approximately 200 μL of cisplatin-loaded hybrid nanoparticles were added with chloroform:methanol mixtures (1:1, v/v) and a vortexed well. This mixture was added to 250 μL chloroform followed by vortexing. Approximately 250 μL of 0.1N HCl solution was added to the mixture and mixed thoroughly before being centrifuged at 10 000 rpm for 5 minutes. The upper aqueous layer containing cisplatin was removed and analyzed by HPLC method (HPLC Shimadzu, Japan) with an anion exchange column Inertsil AX^®^ (250 mm x 4.6 mm, 5 μm) as the stationary phase at room temperature.^40,41^ The mobile phase was composed of ethyl acetate: methanol: Milli-Q water: *N,N*-dimethylformamide (8:40:10:20, v/v) with a flow rate of 1 mL/min. The encapsulation efficiency (EE) and loading capacity (LC) were calculated using equations (1) and (2) respectively:^42,43^

(1) $$Entrapment\;Efficiency(\%)=\frac{cisplatin\;content\;of\;hybrid\;nanoparticle}{total\;added\;amount\;of\;cisplatin}\times 100$$

(2) $$Loading\;Capacity(\%)=\frac{amount\;of\;drug\;encapsulated}{amount\;of\;drug\;encapsulated+total\;amount\;of\;liposomal\;components}\times 100$$

### In vivo biodistribution study of hybrid nanoparticlesloading cisplatin

In order to evaluate the drug biodistribution, 6-week old, female CDF1 mice weighing 20-25 g represented the subjects of this study. They were all purchased from Sankyo Labo (Tokyo, Japan) and treated in accordance with the conditions stipulated by the Guiding Principles for the Care and Use of Laboratory Animals of the Animal Research Committee of Hoshi University. Firstly, the mice were tumor induced by means of xenograft method of C-26 cells, which were transplanted by injecting cell suspension (1 × 10^6^ cells) subcutaneously. After the tumor had reached a size of 100 mm^3^, the mice were divided into five groups of 4-5 subjects.

The samples were administered intravenously twice via tail vein injection at a dose equivalent to 4 mg cisplatin per kg mice per injection. Forty-eight hours after the first injection, a second was administered, twenty-four hours after which the subjects were sacrificed and their blood collected in heparinized tubes. In order to extract the plasma fraction, the blood samples were centrifuged for ten minutes at 9100 G. Blood-free tumor and kidney tissue were taken and weighed. The tissues and plasma were stored at -20^o^C until platinum analysis by graphite furnace atomic absorption spectrophotometry (GF-AAS, Z-8100 Polarized Zeeman, Hitachi, Japan) was performed as previously reported.^26^ Platinum levels were determined by digesting samples with concentrated HNO_3_ and heating them for one hour at 70°C, followed by heating at 120°C overnight to obtain dry samples. These were then added to 0.1 N HCl at an appropriate level of dilution. The GF-AAS analysis program involved three steps: (1) a 40-second drying stage at 80-100^o^C, (2) a 30-second ashing stage at 800°C (3) a 7-second atomization stage at 3000^o^C, followed by cooling. Absorbances were measured at 265.9 nm with a slit bandwidth of 0.4 nm and the sample volume was 30 µL. The results were expressed as µg Pt/mL plasma and µg Pt/g tissue or organ.

### Statistical analysis

All data was in three replicates and presented with the mean ± SD. To evaluate the significance of the differences, the data was analyzed by means of a one way ANOVA test followed by a Tukey’s post hoc test performed using SPSS software v.17.0 with a *P* value <  0.05.

## Results and Discussion

### Physical characterizations of surfactant-modified cisplatin loading hybrid nanoparticles

**Figure 1:**
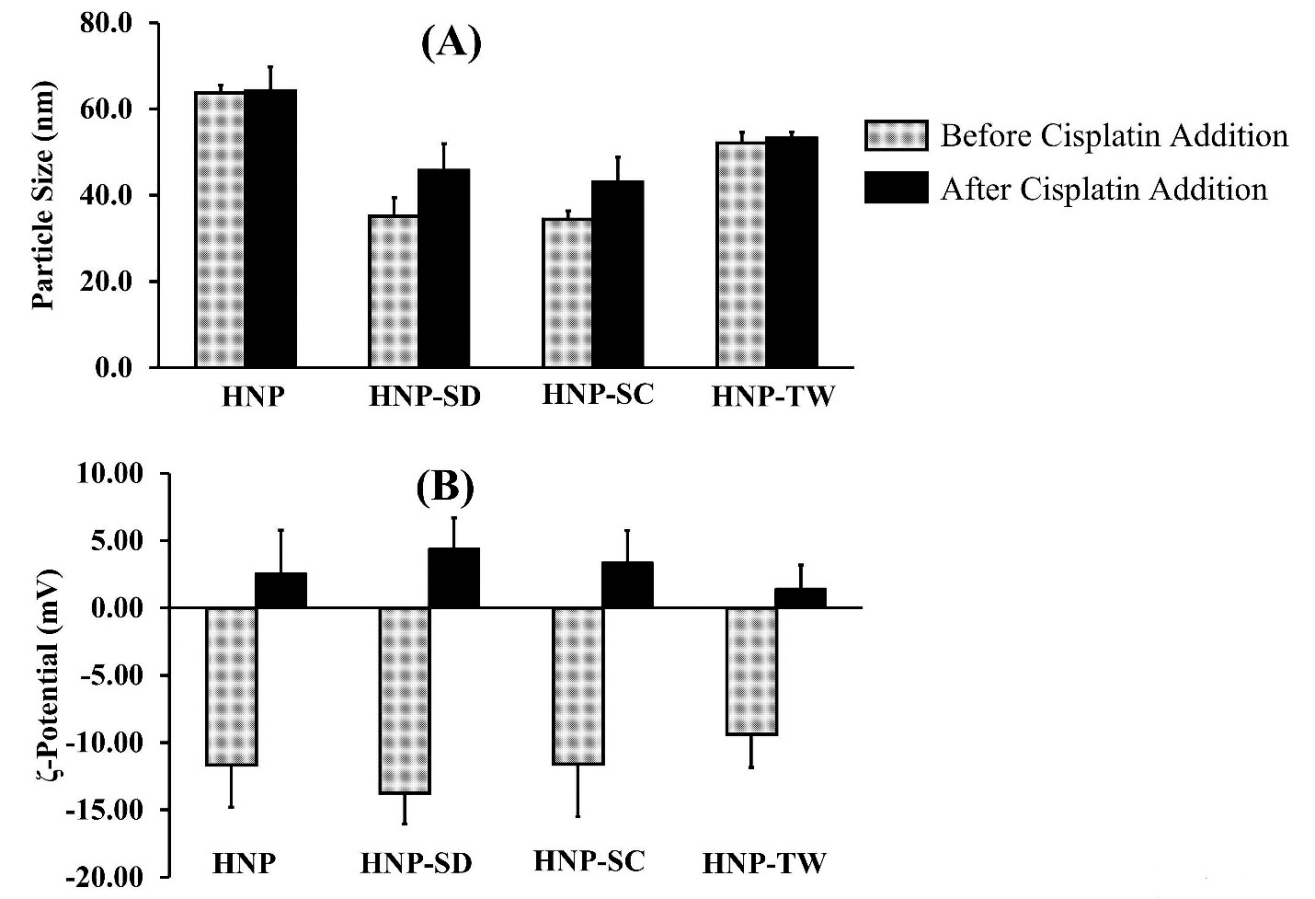
Characterizations of particle size (A) and ζ-potential (B) of hybrid nanoparticles prepared by injection method before and after the addition of cisplatin.

In this study, the hybrid nanoparticles were prepared by injection method. The polymer was prepared by, firstly, dissolving it in the organic solvent and precipitating it in aqueous media to form nanoparticles. The surfactants used in this study were of two types; anionic surfactant, i.e. sodium cholate (SC) and sodium deoxycholate (SD), and non-ionic surfactant (Tween 80/TW). As seen from Figure 1A, all hybrid nanoparticles (HNP) had a particle size less than 100 nm. The addition of surfactants including sodium deoxycholate and sodium cholate to the hybrid nanoparticles, i.e. HNP-SD and HNP-SC respectively, produced slightly smaller particle sizes than HNP. Moreover, as shown in Figure 1B, the ζ-potential of all these hybrid nanoparticles was negative and not significantly different.

The drug loading was achieved simply by mixing the cisplatin solution with an aqueous dispersion of the hybrid nanoparticles matrix in alkaline conditions. The results showed that the addition of cisplatin to hybrid nanoparticles did not significantly affect the particle sizes (Figure 1A). On the other hand, this addition neutralized the ζ-potential of all preparations from a negative charge (around -9.4 to -13.8 mV) to a relatively neutral charge of between 1.4 and 4.4 mV, as shown in Figure 1B.

**Figure 2:**
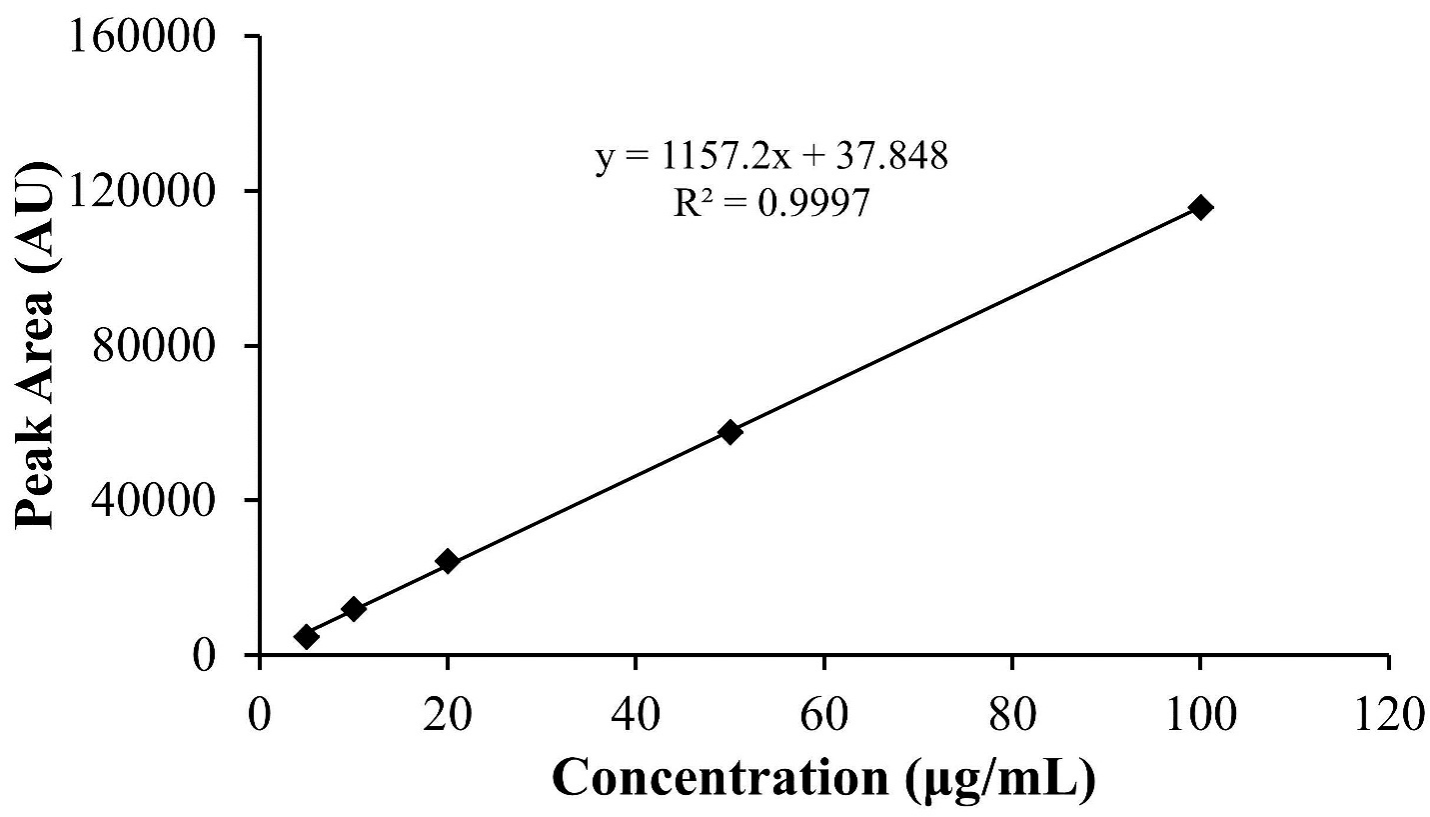
The calibration curve of cisplatin solution in PBS pH 7.4 at the concentration ranges within 5-100 μg/mL measured by HPLC using Inertsil AX^®^ anion exchange column with mobile phase consisted of the mixture of ethyl acetate:methanol:water:N,N dimethylformamide at a ratio of 8:40:10:20, respectively.

**Figure 3:**
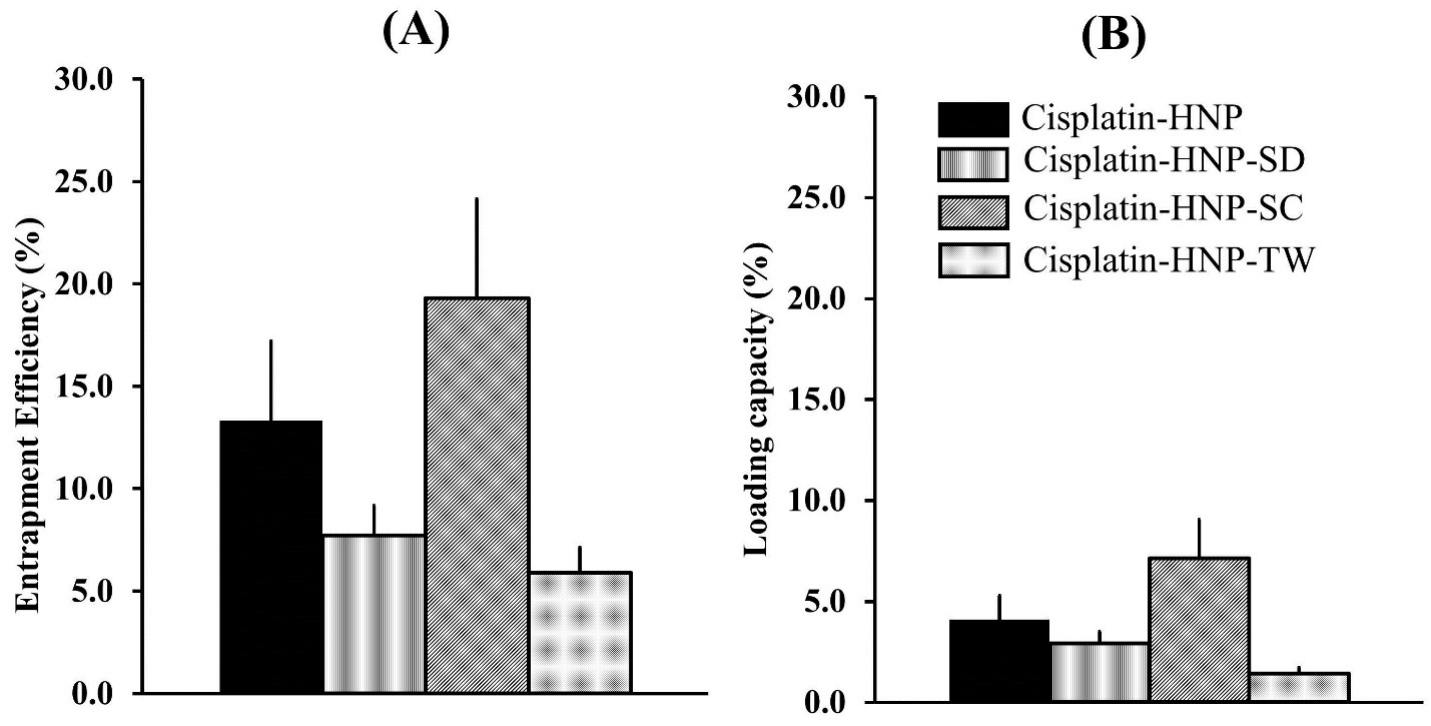
The entrapment efficiency and loading capacity of cisplatin in hybrid nanoparticles without modification of surfactant i.e. cisplatin-HNP and surfactant-modified hybrid nanoparticles, i.e. sodium deoxycholate (Cisplatin-HNP-SD), sodium cholate (cisplatin-HNP-SC), and Tween 80 (cisplatin-HNP-TW). The measurement was analyzed by means of HPLC method.

The amount of cisplatin encapsulated in hybrid nanoparticles were determined by using an HPLC method. The peak areas were then plotted against concentration (Table 2) resulted in a good linearity of calibration curve with coefficient of correlation (R^2^) of 0.9997 over the cisplatin concentration range of 5-100 μg/mL, as seen in Figure 2. The cisplatin entrapment efficiency of cisplatin-HNP, cisplatin-HNP-SD, cisplatin-HNP-SC, and cisplatin-HNP-TW were between 5.4-17.8%, with loading capacity were 1.4-7.1%, as shown in Figure 3A-B. Sodium cholate-modified hybrid nanoparticles (cisplatin-HNP-SC) had the highest drug loading capacity of all the hybrid nanoparticles.

**Table 2:** The peak area of cisplatin standard solution

**Concentration of cisplatin (μg/mL)**	**Peak area**
5	4711
10	11 933
20	24 303
50	57 635
100	115 688

These results correlate closely with those of a previous study which reported that SC coexistence with phospholipid can undergo changes in shape and size due to the formation of mixed micelle and mixed bilayer micelle.^27,39^ However, in this study, hybrid nanoparticles also contained diblock polymer of PEO-b-PMAA whose presence may also contribute to the final hybrid nanoparticle size. Nevertheless, further investigation is required in order to analyze this phenomenon. After the addition of cisplatin, the particle size of hybrid nanoparticles that contained lipid and surfactant were not significantly different. Although the loading of cisplatin has been reported as affecting the cross-linking state of the polymer on micelle formation,^44^ through inter- and intramolecular bonds between carboxylic acid groups of PMAA with active aquatic species of cisplatin,^45^ it was probably insufficient to produce denser particles. Once maximum packed particles had been obtained in the pre-conjugation state, resulted from the spontaneous precipitation of polymer-lipid mixtures, the compaction effects of cisplatin binding to polymer would not produce any further size reduction. On the other hand, the cisplatin loading process generated neutralization of ζ-potential of hybrid nanoparticles complexes as presented in Figure 1B.

### In vivo biodistribution study of cisplatin-loading hybrid nanoparticles

**Figure 4:**
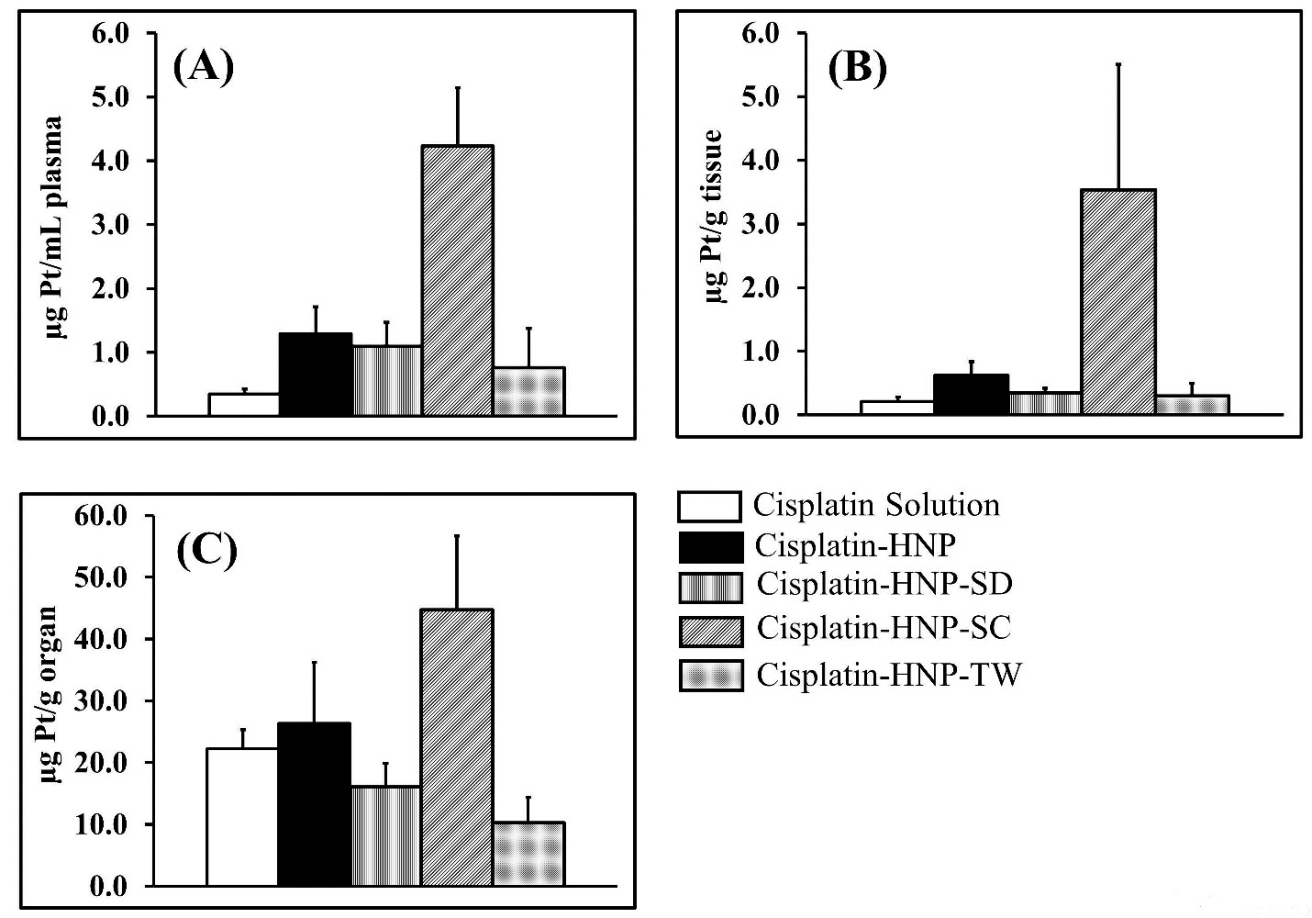
Platinum concentration in plasma (A), tumor (B), and kidney (C) at 24 hours post-intravenous injection of hybrid nanoparticles loading cisplatin with and without surfactant modifications. The samples were administered to C-26 tumor-bearing mice twice, the second injection being given 48 hours after the first with the samples being taken 24 hours later (n=4-5). The platinum levels were analyzed using a GF-AAS method.

An *in vivo* biodistribution study was performed by administering the drugs twice due to a short first-half lifetime.^11^ The data represents the drug concentrations at 24 hours after the second injection. As shown in Figure 4A-B, generally the hybrid nanoparticles, i.e. cisplatin HNP, cisplatin-HNP-SD, cisplatin-HNP-SC, and cisplatin-HNP-TW, produced higher drug concentration in plasma and tumors than the cisplatin solution group. Furthermore, it can be clearly seen from these figures that the addition of sodium cholate to hybrid nanoparticles (cisplatin-HNP-SC) produced the highest drug concentration in plasma, up to ten times higher than that of the cisplatin solution, and high accumulation in tumor tissue among others. This explains how incorporating cisplatin into hybrid nanoparticles (cisplatin-HNP-SC) successfully prolonged the drug circulation in plasma, thus increasing tumor drug accumulation via the enhanced permeation and retention effect.^46^ These results may also correlate with the lipid barrier of hybrid nanoparticles for water diffusion and the cisplatin-polymer (PMAA) conjugation states, However, the effect of sodium cholate in prolonging circulation of cisplatin hybrid nanoparticles, though it affects membrane deformability, is still being investigated. It is known that these three surfactants have different chemical structures that may affect the lipid layered on the hybrid nanoparticle surfaces. Tween 80 contains non-bulky hydrocarbon chains. On the other hand, sodium deoxycholate and sodium cholate have steroid-like structures with differences in the total number of hydroxyl functional groups, which are three and two for sodium cholate and sodium deoxycholate respectively. These structures are bulkier than Tween 80, thus reducing transient hydrophilic hole formation causing rigidity of the lipid layer.^47^ This may limit water permeability across the lipid layer on the hybrid nanoparticles causing cisplatin to leak out. However, this report states that there were no significant differences in lipid rigidity between sodium cholate and sodium deoxycholate, while in this study sodium cholate produced more stable nanoparticles than sodium deoxycholate – a phenomenon requiring further evaluation.

On the other hand, as shown in Figure 4C, elevated drug levels in the kidney were observed in this group generating two-fold higher platinum levels than in the cisplatin solution treatment group. The long circulated cisplatin-HNP-SC probably releases cisplatin which experiences a biphasic elimination phase with the late phase within 2-3 days,^11^ in slow mode, thus leading to the accumulation of high cisplatin levels in the kidney as the excreting organ.

## Conclusion

The hybrid nanoparticles loading cisplatin were prepared with the addition of surfactants. Sodium Cholate modification had successfully reduced the particle size of the cisplatin hybrid nanoparticles and improved plasma drug circulation as well as the tumor drug accumulation of cisplatin. However, further investigation is required to evaluate the effects of sodium cholate on the biological barrier with the presence of diblock polymer as the core of cisplatin hybrid nanoparticles.

## Ethical Issues

The animal experimental investigation employed in this study complied with the Guiding Principles for the Care and Use of Laboratory Animals as established by the Animal Research Committee of Hoshi University.

## Conflict of Interest

The author declare no conflicts of interest or financial interests in any product or service mentioned in this article, including grants, employment, gifts, stock holdings, honoraria, consultancies, expert testimony, patents, and royalties.

## Acknowledgments

The authors wish to express their gratitude to Dr. Kumi Kawano, Prof. Yoshiyuki Hattori, and Prof. Etsuo Yonemochi for their support of this study conducted at the former Fine Drug Targeting Laboratory, Hoshi University, Tokyo, Japan.
